# Anti-*Anisakis* IgE Seroprevalence in the Healthy Croatian Coastal Population and Associated Risk Factors

**DOI:** 10.1371/journal.pntd.0002673

**Published:** 2014-02-06

**Authors:** Ivona Mladineo, Vedran Poljak, Victoria Martínez-Sernández, Florencio M. Ubeira

**Affiliations:** 1 Institute of Oceanography and Fisheries, Laboratory of Aquaculture, Split, Croatia; 2 Health Ecology Department, Croatian National Institute of Public Health, Zagreb, Croatia; 3 Department of Microbiology and Parasitology, Faculty of Pharmacy, University of Santiago de Compostela, Santiago de Compostela, Spain; Uniformed Services University, United States of America

## Abstract

**Background:**

The main objective of the study was to determine the degree of sensitization to *Anisakis* spp. antigens in healthy coastal population of Dalmatia given the high thermally unprocessed fish intake rate present in this area, suggested as a significant risk factor for anisakiasis. We performed a monocenter, cross-sectional pilot study stratified by geographic area of residence, conducted at the County secondary healthcare provider Medicine-biochemical Laboratory in Split (Croatia), from November 2010 till December 2011, on 500 unpaid volunteer subjects undergoing routine blood analysis and belonging to the south coast of the Adriatic Sea.

**Methodology/Principal Findings:**

We studied the IgE seroprevalence to *Anisakis* spp. Ani s l and Ani s 7 allergens by indirect ELISA in healthy subjects, which were selected at random in the region of Dalmatia (Southern Croatia), among islands, coastal urban and inland rural populations. In order to detect possible cross-reactivity to other human helminthes, serum samples were tested also for the presence of IgG antibodies to *Ascaris lumbricoides* and *Toxocara canis*. The overall and coastal *Anisakis* seroprevalences for the sampled population were 2% and 2.5%, respectively. The logistic univariate regression analysis confirmed that regarding anti-*Anisakis* IgE seroprevalence, raw fish intake, daily fish intake, homemade origin of fish dish and occupational contact (professional, artisanal or hobby contact with fishery or fish industry) were risk factors associated to *Anisakis* spp. sensitization, but neither of the variables was exclusive for a particular seropositive population. Also, a significant difference was observed between seropositive and seronegative subjects that had stated allergy or symptoms associated with allergy (atopic dermatitis, asthma or rhinitis) in their previous history.

**Conclusions/Significance:**

Being the first in Croatia, our study underlines the necessity of incorporating *Anisakis* spp. allergens in routine hypersensitivity testing of coastal population.

## Author Summary

Anisakiasis is a zoonosis induced by infection with the *Anisakis* third-stage larvae, contracted through consumption of thermally unprocessed or lightly processed seafood. Its diagnosis is difficult to suspect in countries where the illness was not previously reported, where it is infrequent, or in the cases of subclinical infections. Therefore, it is of great relevance to conduct epidemiological studies to assess the seroprevalence of anti-*Anisakis* IgE in populations where this zoonosis is more probable. A cross-sectional pilot study was performed on 500 subjects undergoing routine blood analysis and belonging to the south coast of the Adriatic Sea. The results showed that IgE sensitization to *Anisakis*, tested by indirect ELISA using recombinant Ani s 1 or Ani s 7 allergens, reached 3.5% in the population of higher fish consumers (islands). All *Anisakis* positive subjects were high fish consumers, mostly of raw and homemade thermally unprocessed fish prepared in the traditional manner. Most of them reported professional or hobby occupational contact with fishery or fish industry. We demonstrated that in coastal Croatian populations there is a relevant prevalence of *Anisakis* infections, mainly related to the ingestion of home-made raw fish, underlining the necessity to carry out a wider epidemiological study of *Anisakis*-induced allergy.

## Introduction

Anisakidosis is a zoonotic disease caused by members of the nematode family Anisakidae, whereas anisakiasis ( = anisakiosis) is caused by members of the genus *Anisakis* [1]. It is considered one of the most significant emerging food-borne diseases [2], [3], [4] because the more stringent measures regarding conservation of sea mammals, which are the final hosts, and the acquisition of new gastronomic habits throughout Europe [5] have led to an increase in *Anisakis* infection rate within paratenic fish host and human population. More medical consciousness of the disease and more detailed clinical examinations have enhanced the number of diagnosed cases in humans [6], although it is still a misdiagnosed and underestimated entity in Mediterranean. *Anisakis* third-stage infective larvae are contracted through consumption of thermally unprocessed or lightly processed traditional seafood: sushi and sashimi in Japan [7], tuna or sparid carpaccio, marinated, salted or pickled anchovy in Mediterranean [8], [9], [10], smoked or fermented herrings (*maatjes*) in Netherlands [11], dry cured salmon (*gravlax*) in Norway, raw salmon (*lomi lomi*) in Hawaii or ceviche in South America [12]. Depending on the site of infection, the parasitization by live *Anisakis* third-stage larvae can elicit gastric, intestinal or ectopic anisakiasis [13]. Gastric anisakiasis is characterized by epigastric pain, nausea and vomits after a short period of 1–12 h postingestion of live *Anisakis* larvae [1]. In the intestinal form, abdominal pain is also the predominant symptom, but the incubation period may be delayed until 48–72 h postingestion [14].

A relevant number of patients with gastric anisakiasis can present associated allergic symptoms ranging from urticaria to anaphylactic shock, and this clinical entity was named gastroallergic anisakiasis [15], [16]. The allergic symptoms may predominate over gastrointestinal manifestations, which explains why many of these patients are attended by allergologists instead of digestive specialists. Furthermore, most *Anisakis* infections are subclinical [8], [17], and this condition can only be detected using immunological tests [18]. *Anisakis* infections were also related to the increased risk of upper gastrointestinal bleeding in patients consuming nonsteroidal anti-inflammatory drugs [17] and neoplastic and carcinogenic changes in human intestinal system [19], [20].

The allergic aspects of *Anisakis* infections have been extensively studied in the past decade, mainly in Spain [6], [16], [21], where hundreds of cases of allergy to *Anisakis* have been reported since 1995 [6], [9], [18], [22], [23]. These results have recommended to carry out serological studies in other Mediterranean populations, both healthy or with food allergies in anamnesis to understand the relevance of *Anisakis* infections in Europe [24], [25]. In south coastal part of the Adriatic Sea, Croatian population has been traditionally engaged in preparation of home-made thermally unprocessed fish, mostly pickled, marinated, salted anchovy (*Engraulis encrasicolus*) and sardine (*Sardina pilchardus*), or salted damselfish (*Chromis chromis*), as a particular ethnically recognized dish, geographically limited to Island of Korčula. This is very important to consider in relation to *Anisakis* infection in humans because the elevated consumption of such dishes as national staple food correlates with the peak of tourist season in summer.

The aim of this pilot study was to assess the seroprevalence of anti-*Anisakis* IgE antibodies in coastal healthy population, where infection is feasible given the high rate of undercooked anchovy consumption and anchovy's high infection rate with *A. pegreffii* [26]. Further, we aimed to pinpoint the extent to which thermally unprocessed fish intake, home-made or marketed seafood contribute to the risk of *Anisakis* sensitization applying a logistic regression analysis to data collected through an anonymous questionnaire.

## Methods

### Study design and patients

This was a monocenter, cross-sectional pilot study stratified by geographic area of residence, and conducted at the County secondary healthcare provider Medicine-biochemical Laboratory in Split (Croatia), from November 2010 till December 2011. Split is the capital town of Split-Dalmatia County (455000 inhabitants), with a population of 200000 inhabitants (second largest in Croatia), and a major administrative, commercial, touristic and transit junction. The sample size was a priori determined by use of the Epidat 4.0 software package (http://dxsp.sergas.es) assuming the *Anisakis* sensitization prevalence to be similar or less than 13.5%, which is the mean prevalence for various Spanish communities, as determined using the same procedure employed in the present study [9], [17]. The estimated sample size for a population of 455000 inhabitants was 498, considering a 3% absolute precision, and a 95% confidence level. The sample size was rounded up to 500 participants which were distributed by stratified random sampling in three groups of 200, 200 and 100 participants according to residency in one of three geographic subareas considered, respectively: islands population (50000 inhabitants), assumed as high fish eaters (group A); coastal urban population (250000 inhabitants), assumed as medium fish eaters (group B) and inland rural population (70000 inhabitants), assumed as low or non-fish eaters (group C). Northwest mountain population of Split-Dalmatia Country (85000 inhabitants) was excluded from analysis because it does not belong to the historical Dalmatia region, a term informally used in practice nowadays. Initially, health providers recruited healthy subjects among individuals that underwent systematic medical examination for different purposes (working permit, driving license, military training and routine cholesterol control) over one year. All eligible participants were healthy adults, aged 25 or over, with residency in one of the three aforementioned geographic subareas. Subjects were excluded if they did not reside in the investigated area, were under 25 years of age or had acute or chronic infectious disease symptoms at the time of blood sampling. Approximately, 20% of eligible patients agreed to participate in the study. Signed consent and personal contact information were obtained from all subjects prior to the extraction of 5 ml of blood. Serum was separated by centrifugation at 3000 rpm for 10 minutes, and stored at −20°C. Those eligible participants were included in the software SimDis (Monte Carlo simulation; http://www.izor.hr/web/guest/simdis), which generated a list of 500 random numbers per area of residence. Independent healthcare workers distributed 500 sealed, non-transparent envelopes to each group (A, B, C) at the healthcare provider, of which only a half contained an anonymous food frequency questionnaire regarding personal, occupational details and fish-eating habits (see supporting Figures S1, S2 and supporting Text S1). The person that generated the random sampling was different from the healthcare providers that distributed the envelopes. The questionnaire included items related to: 1) personal, occupational and health details (gender, age, contact with fishery or fish industry, food allergy or symptoms associated with allergy: atopic dermatitis, asthma or rhinitis); 2) fish consumption preferences (raw fish, frozen, grilled, cooked, canned); 3) consumption of thermally unprocessed fish (raw fish) including method of preparation (raw, salted, marinated, sushi); 4) frequency of consumption (daily, several times a week, once a week, rarely, never); and 5) origin of fish for consumption (home-made, retail, restaurant). Only eligible participants that had envelopes with the questionnaire were considered for inclusion. Finally, the eligible participants with coastal urban (200 subjects), island (200 subjects) and inland rural (100 subjects) residency that responded first (of the 250 subjects in each group that received an envelope with the questionnaire) were analyzed for *Anisakis* seropositivity. Outcome assessors and data analysts were kept blinded to the distribution of the participants in the three groups.

### Serological determinations

IgE sensitization to *Anisakis* spp. was tested in indirect ELISA using recombinant Ani s 1 and Ani s 7 allergens as target, a method that has been reported to be highly specific and sensitive, and proposed as the gold standard for serodiagnosis of human *Anisakis* infections [18], [27]. In this assay each serum (100 µl, undiluted) was tested in three individual wells containing, respectively, Ani s 1, Ani s 7, or no antigen. The results, expressed as optical densities (OD) at 492 nm, were calculated by subtracting from the OD value given by each allergen, the OD value produced by the same serum in the absence of allergen. The cut-off OD values for Ani s 1 (OD = 0.09) and for Ani s 7 (OD = 0.05) were previously calculated using a collection of negative sera (200 sera for Ani s 1 and 561 sera for Ani s 7) from Spanish healthy blood donors aged 18 to 65 years [18], [28]. For such calculations, the mean OD obtained with the negative sera plus 4 SD was considered. As previously reported [18], a serum was classified as truly positive when it tested positive to Ani s 1, Ani s 7, or both allergens. Considered individually, there is agreement that both allergens are 100% specific [18], [29], [30], but Ani s 1 proved to be less sensitive (sensitivity = 61.1%; 95% confidence interval, CI 54.07–68.15%) than Ani s 7 (sensitivity = 93.94%; 95% CI 90.36–97.52%) [18].

In order to confirm that the specificity of the Ani s 1/Ani s 7 serological test does not change when testing sera from non-Spanish populations, in parallel with anti-*Anisakis* IgE determinations we tested the Croatian sera for the presence of IgG antibodies to other two related ascarids, *Toxocara canis* and *Ascaris lumbricoides*. Such determinations were done using a commercial ELISA for detection of IgG antibodies to *T. canis* and *A. lumbricoides* (Novatec Immunodiagnostica GmbH, Germany) according to the manufacturer's recommendations. Three sets of serum samples were considered: i) individual sera testing positive for anti-*Anisakis* IgE antibodies against the allergens indicated above (n = 10), ii) pooled samples (19–20 sera pooled in each sample) from islands (n = 10) and coastal urban (n = 10) populations, testing negative for *Anisakis*, and iii) pooled sera (10 sera pooled in each sample) from the inland rural population (n = 10). Briefly, horseradish peroxidase labeled protein A conjugate was added after incubation of sera in the 96-well microtiter plate coated with antigen from *T. canis* and *A. lumbricoides* and the reaction was visualized using 3,3′,5,5′-tetramethylbenzidine substrate. Absorbance at 450 nm was read using an ELISA microwell plate reader with the cut-off as the mean absorbance value of the cut–off control determinations. Assay results were presented in NovaTec-Units (NTU) as sera mean absorbance value ×10/cut-off = NTU (cut-off value: positive >11 NTU) [31], [32].

### Statistical calculations

The strength of association between dependent (IgE seropositivity to *Anisakis* spp.; yes/no) and independent variables 1) raw fish consumption; 2) daily fish consumption; 3) several times per week consumption; 4) home-made origin of fish dish; 5) occupational contact; 6) age >50 (yes/no); was inferred by univariate logistic regression analysis using software package Stata/IC, version 11.2. Both dependent and independent variables were dichotomous variables. Odds ratio (OR) values were considered statistically significant if the 95% CI did not include 1. Anti-*Anisakis* seroprevalence and its Fisher's confidence intervals were calculated by WinPepi [33].

The correlation between antibody presence and subject age was performed using Phi coefficient (r_φ_). Fisher's exact test (two-tailed) was used to determine the difference in prevalence of sensitization between the genders, as well as to detect any possible false positive result in the IgE determinations due to cross-reactivity with infections caused by other ascarid nematodes. *p*-values<0.05 were considered to be statistically significant in all the analyses.

### Ethics statement

This research was approved by the Ethics Committee of the Croatian National Institute of Public Health No: 001- 41/1-11. All patients included have given their written informed consent.

## Results

### Sample characteristics and eating habits

The age and gender distribution of the sample is shown in Table 1. The mean age of the 500 subjects participating in the study was 58.1 years. The 51.6% of the population in the sample consisted of males (n = 258), whereas 48.4% were females (n = 242). The mean age of all the *Anisakis* spp. positive subjects (n = 10) was 63.1 (range 47–77). No significant correlation was found between antibody presence and subject age (Phi coefficient r_φ_ = 0.1846; *p* = 0.3205) or any difference in the prevalence of sensitization between genders (Fisher's exact two-tailed test *p* = 1.000).

**Table 1:** Characteristics of the three groups considered in the study.

	Age		Males		Females		Raw fish consumption		Home-made origin of fish dish		Occupational contact	
Geographic area	Mean	Range	n	%	n	%	n	%	n	%	n	%
Group A (n = 200)	65.3	48–87	10	54.5	91	45.5	168	84	161	80.5	129	64.5
Group B (n = 200)	56.3	36–91	103	51.5	97	48.5	62	31	22	11	31	15.5
Group C (n = 100)	52.4	25–84	46	46	54	54	0	0	0	0	0	0
Total (n = 500)	58.1	25–91	258	51.6	242	48.4	230	26	183	39.2	160	32

Group A: islands population; Group B: coastal urban population; Group C: inland rural population.

The analysis of the questionnaire showed that in islands (group A), predominated the answers “daily” and “several times a week fish consumption” (26.5% and 57.5%, respectively) while in coastal urban population (group B), the answers “once a week” and “several times a week fish consumption” were more frequent (61.5% and 25.5%, respectively). As expected, in the inland rural population (group C), predominated the answers “rarely” and “never” (51% and 27%, respectively) (Supporting Figure S1). The results (Table 2) show that all *Anisakis* positive subjects were high fish consumers (daily or several times a week), while most of them (9/10) reported eating raw and home-made thermally unprocessed fish prepared in the traditional manner. Also, most of seropositive subjects (8/10) reported professional, artisanal or hobby occupational contact with fishery or fish industry.

**Table 2:** Characteristics and personal habits of *Anisakis* spp. seropositive subjects.

Group	Age	Gender	Allergy symptoms	Daily or several times a week fish consumption	Raw fish consumption	Home-made origin of fish dish	Occupational contact	Ani s 1	Ani s 7
A	72	F	_	+	+	+	+	0.006	**0.090**
A	75	F	_	+	+	+	_	0.008	**0.055**
A	73	M	_	+	+	+	+	0.007	**1.872**
A	60	M	+	+	+	+	+	**0.368**	**1.291**
A	52	F	+	+	+	+	+	0.006	**0.306**
A	52	F	_	+	_	+	+	0.001	**0.164**
A	77	M	_	+	+	+	+	**1.924**	**1.323**
B	66	M	_	+	+	+	+	**0.558**	**0.384**
B	47	F	+	+	+	_	_	0.005	**0.304**
B	57	M	_	+	+	+	+	0.006	**0.141**

A: islands population; B: coastal urban population; F: female; M: male. The values in Ani s 1 and Ani s 7 columns show ODs obtained in ELISA for each serum (positives in bold). Cut-off values were OD = 0.09 for Ani s 1 and OD = 0.05 for Ani s 7.

### Seroprevalence of anti-*Anisakis* IgE antibodies

Of the 500 sera tested for anti-Ani s 1 and Ani s 7 IgE antibodies by indirect ELISA, 10 tested positive (2%) (Table 2). Seropositive subjects included 5 men and 5 women aged 57–77 and 47–75 years, respectively. Of the 10 seropositive subjects, 7 were from the islands population (group A; prevalence = 3.5%, Fisher's exact 95% CI 1.42–6.45) and 3 from the coastal urban population (group B; prevalence = 1.5%, Fisher's exact 95% CI 0.31–4.32), which represents a mean seroprevalence of 2.5% (95% CI 1.1–3.9) for the whole sampled coastal population. Fisher's exact CI for rural population (group C; n = 100), where no seropositive subjects were detected, was 0.0–3.62%. Comparing the response to the two *Anisakis* allergens, 3 subjects were positive for Ani s 1, while all were positive for Ani s 7.

### Seroprevalence of IgG antibodies to other related helminthes

**Figure 1:**
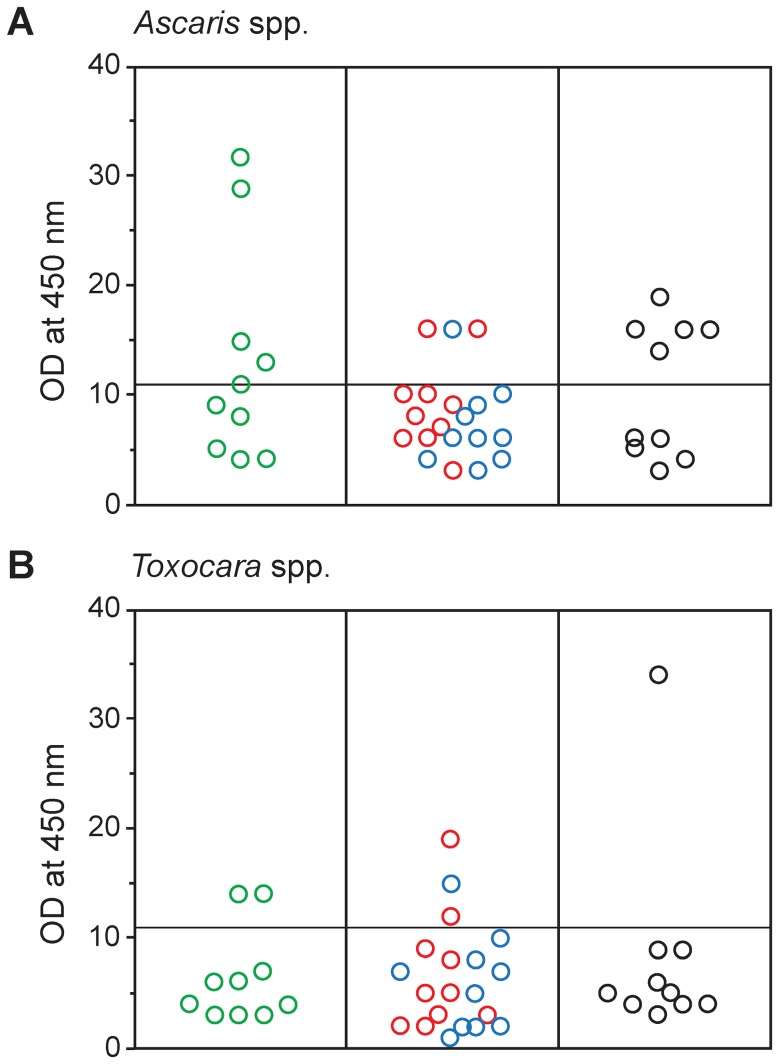
Anti-*Ascaris/Toxocara* IgG response. Anti-*Ascaris* spp. IgG (A) and anti-*Toxocara* spp. (B) NTU values in the population of *Anisakis* positive sera (green circles, n = 10 individual sera), and the population of *Anisakis* negative sera comprising: i) islands (red circles; n = 10 pooled sera) and coastal urban populations (blue circles; n = 10 pooled sera), and ii) inland rural population (black circles; n = 10 pooled sera). IgG levels are expressed as OD at 450 nm in NTU. Horizontal bars represent cut-off values for each parasite (positive *Ascaris* spp. >11; *Toxocara* spp. >11).

Individual serum samples with IgE antibodies to *Anisakis* spp. (n = 10; groups A and B), together with 20 pooled samples of the seronegative sera from coastal populations (n = 390; groups A and B) and 10 pooled sera from the inland rural population (n = 100; group C) were tested for IgG antibodies to *A. lumbricoides* and *T. canis* (Figure 1). Among the *Anisakis*-positive subjects, 4 individual sera were positive for *Ascaris* spp. and 2 individual sera were positive to *Toxocara* spp. (40% and 20%, respectively). Considering the *Anisakis*-negative subjects from coastal populations (n = 390; groups A and B), 3/20 pooled sera were positive to *Ascaris* and another 3 pooled sera were positive to *Toxocara* (15% in both cases). Finally, in the inland rural population (n = 100), 5/10 pooled sera were positive to *Ascaris* and 1 pooled sera was positive to *Toxocara* (50% and 10%, respectively). No statistically significant NTU differences were found comparing *Anisakis*-positive and *Anisakis*-negative samples in these analyzed groups of sera considering either seropositivity to *Ascaris* (Fisher exact two-tailed test; p = 0.1605), or *Toxocara* (Fisher exact two-tailed test; p = 0.6269). In fact, only a single *Anisakis-*positive serum also tested positive on both *Ascaris* and *Toxocara* ELISA (29 and 14 NTU, respectively).

### Univariate logistic regression analysis

Comparing the seropositive (10 subjects) and seronegative populations (490 subjects), the univariate logistic regression analysis confirmed that raw fish intake (OR = 10.95; *p* = 0.024), daily fish intake (OR = 10.54; *p*<0.0001), home-made origin of fish dish (OR = 16.34; *p* = 0.0008) and occupational contact (OR = 8.89; *p* = 0.006) were risk factors associated with *Anisakis* spp. sensitization. Other details are shown in Table 3.

**Table 3:** Odds ratio of the risk factors associated to seropositivity to *Anisakis* spp.

Subject habits	Raw fish consumers	Daily fish consumers	Home-made origin of fish dish	Occupational contact
Seropositive	9/10 (90%)	6/10 (60%)	9/10 (90%)	8/10 (80%)
Seronegative	221/490 (45.1%)	61/490 (12.44%)	174/490 (35.51%)	152/490 (31.02%)
All	230/500	67/500	183/500	160/500
*p* value	0.024	<0.0001	0.0008	0.006
OR	10.95	10.54	16.34	8.89
CI 95%	1.37–87.17	2.89–38.44	2.05–130.08	1.86–42.38

Regarding risk factors related to allergy, we have observed that 42/500 (8.4%) of the subjects reported a history of food allergy or symptoms associated with allergy, while 3/10 (30%) seropositive subjects reported allergy history. There was a significant difference between seropositive and seronegative subjects (Fisher's exact two-tailed test *p* = 0.0437) reporting history of allergy symptoms with OR = 4.95 (95% CI 1.232–19.93).

## Discussion

In this study we present the first epidemiological data on *Anisakis* infections in Croatia. Diagnosis of anisakiasis is difficult to suspect in countries where the illness was not previously reported, where it is infrequent, or in the cases of subclinical infections. These aspects point out the relevance of conducting epidemiological studies to assess the seroprevalence of anti-*Anisakis* IgE in the high-risk coastal population, where this zoonosis is more probable. Our data showing an anti-*Anisakis* IgE seroprevalence in healthy subjects of 1.5% in coastal urban population and 3.5% in islands population from Croatia indicated the existence of a relevant number of subclinical infections among general adult population. However, these numbers might be underestimated because patients from allergy services were not included in our study. In fact, a previous study reported an increase of seroprevalence from 11.7% to 16% when considering allergic patients [9].

In our study we have observed that Ani s 1 and Ani s 7 allergens were differentially recognized by positive subjects, which seems to be more tightly related to the immunodominance of the Ani s 7 allergen than to its life span in circulation. Whilst both belong to the major allergen category [27], and antibodies to Ani s 1 are detectable in sera for a longer time than Ani s 7 [18], there are several studies demonstrating that the number of patients recognizing Ani s 7 is higher than for Ani s 1 [18], [27], mainly in the group of patients having chronic urticaria [27]. This highlights its significance as the main target allergen for serodiagnosis of human anisakiasis.

The analysis of the serological data presented in this work also seems to indicate that infections by the three antigenically related nematodes *Anisakis*, *Ascaris* and *Toxocara* [34], [35] coexist in the Croatian population. This fact gave us the opportunity to investigate the specificity of the serological tests used in the study. From a statistical point of view it would be expected that if some antigens used in the different tests were cross-reactive, a significant proportion of seropositive subjects would test positive by more than one test. In addition, whether the *Anisakis* test was not totally specific, it would be also expected that positive cases to *Anisakis* were present in the population not consuming raw or undercooked fish, where infection by *Ascaris*/*Toxocara* was present. In the present study we have observed that 60% (6/10 cases) of sera testing positive to *Anisakis* were also positive to *Ascaris*, *Toxocara* or both, which is greater than would be expected by chance, and therefore a possible indicator of cross-reactivity. This might be the case of a single serum that tested positive to *Ascaris* and *Toxocara* in the group of positive sera to *Anisakis*. However, since there were no positive cases to *Anisakis* in the inland rural population where cases of *Ascaris/Toxocara* were present, it seems that the release of cross-reactive antigens during *Anisakis* infections produces false positive results in the ELISA tests to *Ascaris/Toxocara* but not in the inverse way. This is logical taken into account that, unlike the *Ascaris/Toxocara* tests that use a pool of antigens and have only 95% specificity, the *Anisakis* test used in this study is based on the use of recombinant antigens that proved to be 100% specific for anti-*Anisakis* IgE determinations in previous studies carried out in Spain [9], [18], [30], [36].

*Ascaris* and *Toxocara* infections are relatively frequent in Croatia; *T. canis* seroprevalence in the asymptomatic children with eosinophilia was reported to reach 31% [37], while *Ascaris* spp. induced more acute non-allergic clinical manifestations [38]. Recent official data from the Croatian National Institute of Public Health reported prevalences of 29.45% for *A. lumbricoides* and 21.7% for *Toxocara* spp. in Croatian population during a two-year period (2010–2011; M. Sviben, personal communication).

Through univariate logistic regression analysis we have confirmed that raw fish intake, daily fish intake, and home-made origin of fish dish, were the main risk factors associated to *Anisakis* spp. sensitization. Similarly, in Madrid population, very high consumption of *boquerones* (5.49 g/person/day) and high infection rate in anchovy population contribute to the observed high anti-*Anisakis* seroprevalence [9]. The difference between the prevalence of 3.5% observed in islands and 1.5% in coastal urban populations seems to be due to differences in fish consumption between both areas. Likewise, home-prepared dishes (undercooked or lightly grilled) might also increase the risk of infection, as reported earlier [8], [39]. However, given the overlap between consumption habits of the ten seropositive subjects in this study, where neither of the variables was discriminative for a specific group of subjects, it is unfeasible to pinpoint which of the risk factors is primarily associated with *Anisakis* seropositivity. Instead, it might be that all of them, together with other factors that affect larvae survival, as variations in the prevalence and intensity of infection in the fish, and the manner in which fish dishes are prepared, contribute to the probability of infection of consumers [8].

High variations in *Anisakis* seroprevalence are also frequently observed even among different regions of the same country [6], [40]. In the case of Spain, a mean prevalence of 15.4% in adult population from Madrid was recently observed using the same technique as in the present study [17]. However, this percentage was only 0.4% [8] and 1.5%, respectively, in blood donors and general adult population (unpublished results) from Galicia (NW Spain). Authors related these variations with a different tradition in consumption of pickled anchovies (*boquerones*) that is high in center [9], south [39] and north of Spain [40], but low in Galicia [8], in spite of the fact that this latter region has one of the highest consumption of marine fish in Spain [8], [41]. Similarly, a very low prevalence of *Anisakis* infections and high fish intake was recently reported by Lin et al. [42] in Bergen (Norway), where consumption of raw or undercooked marine fish seems to be infrequent. In Croatia the mean fish consumption is relatively low (8.5 kg/year per capita), but it is extremely biased towards coastal and islands areas that have much higher consumption compared to inland rural area (with <1 kg/year [43]). This very low fish consumption and no raw fish intake in the rural area explains why all cases of seropositive patients that occurred in our study were from coastal and islands populations, and reinforces previous studies showing that home-made raw fish is the main recognized risk factor for *Anisakis* infections/allergy [8], [9]. Like in our study, the AAITO-IFIACI *Anisakis* Consortium [25] has reported that marinated home-prepared anchovies, frequently consumed, probably represent the most common food that causes *Anisakis* sensitization in Italy along the Adriatic west coasts. Along Croatian eastern side of the Adriatic Sea the traditional method of preparing raw fish varies according to regions and islands, but the methods adopted in fish manufacturing ensure killing of *Anisakis* spp. larvae [44]. This is important given the high frequency of *Anisakis* spp. prevalence in certain species of small pelagic fish [26].

Contrary to previous studies [39], our logistic analysis revealed that the risk has no tendency to increase with age (over 50 years), which may be due to the fact that there were no subjects in the range between 25 and 47 years in the islands population. Such underrepresentation of a “younger” age class might have introduced a bias in analysis of correlation towards underestimation of correlation between seroprevalence and age in our study. At the same time, it might have overestimated the overall seroprevalence in islands population, being enhanced by a larger number of older subjects that had a greater possibility of having more exposures to *Anisakis* allergens. This should be taken into consideration during interpretation of the results, as well as when designing similar studies in limited island areas.

From all subjects engaged in the sampling, 8.4% reported history of food allergy or symptoms associated with allergy while this percentage increased to 30% (3/10) in the seropositive population to *Anisakis* (Table 2). The comparison between sensitized versus non-sensitized subjects showed significant difference in reporting food allergy or symptoms associated with allergy, with OR = 4.95. This strongly suggests that *Anisakis* infections are responsible for some of the allergic reactions in the subjects of this study, confirming previous results [16] and supporting the need of introduction of routine *Anisakis* hypersensitivity tests in the risk population.

*Anisakis* spp. was also related with occupational seafood allergy [40], induced by the contact of parasite allergens with skin or respiratory epithelium [45]. In this sense, Italian authors reported that *Anisakis* larvae represent a potential occupational risk in fishermen and workers assigned to fish processing and sale, with specific anti-*Anisakis* IgE detected in 20.2% of the studied population [46]. Similarly, recent research in Croatia described that chronic respiratory symptoms associated with occupational asthma were significantly dominant in fish processing workers compared to controls [47]. To know whether *Anisakis* spp. is a risk factor in occupational allergy is important because many people in the Mediterranean, Croatia included, are engaged in fishery and fish processing or retail industry and as such need to be protected. In our study we have observed significant differences considering the variable “occupational contact” among seropositive and seronegative populations (Table 3) that need to be taken into account for future allergologic studies as well as in routine testing.

In summary, our study demonstrates that, like in other Mediterranean countries, in coastal Croatian populations there is a relevant prevalence of *Anisakis* infections, which were mainly related to the ingestion of home-made raw fish (i.e., anchovies). These data are of interest for allergologists and health authorities in order to carry out a correct diagnosis of *Anisakis*-induced allergy, introduce *Anisakis* in routine hypersensitivity testing and to prevent new *Anisakis* infections.

## Supporting Information

Figure S1 Questionnaire about frequency of fish consumption (daily, several times a week, once a week, rarely, never). A: islands population; B: coastal urban population; C: inland rural population.
(TIFF)

Figure S2 Questionnaire about fish cooking habits. A: islands population; B: coastal urban population; C: inland rural population.
(TIFF)

Text S1 Questionnaire layout used in this study.
(DOC)
