# Supplementary figures and images for: Anti-Anisakis IgE Seroprevalence in the Healthy Croatian Coastal Population and Associated Risk Factors
Source: PLoS Negl Trop Dis. 2014 Feb 6;8(2):e2673. doi: 10.1371/journal.pntd.0002673 (PMC3916232; doi:10.1371/journal.pntd.0002673)

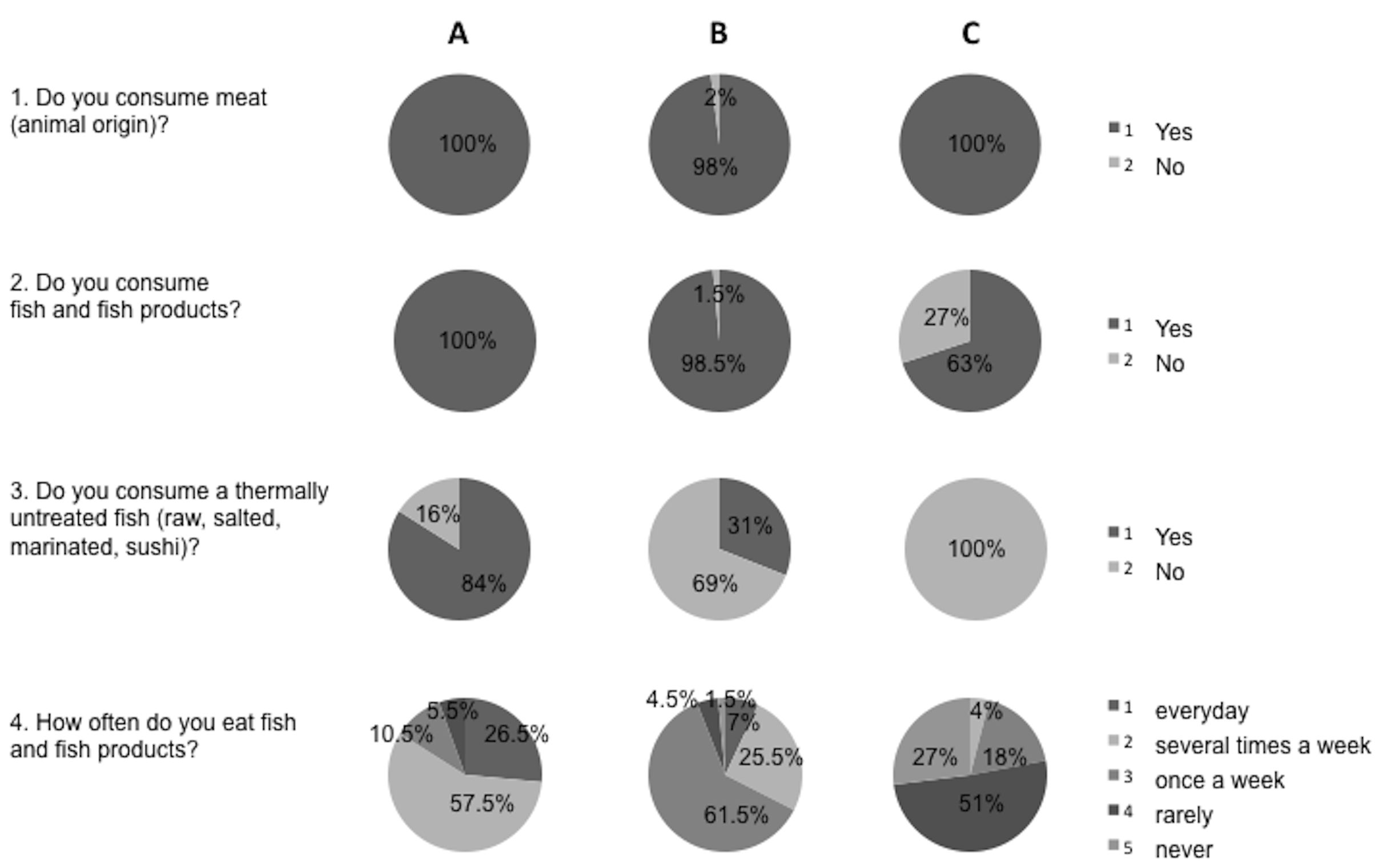

Supplement: Figure S1 — Questionnaire about frequency of fish consumption (daily, several times a week, once a week, rarely, never). A: islands population; B: coastal urban population; C: inland rural population. (TIFF) [file pntd.0002673.s001.tiff]

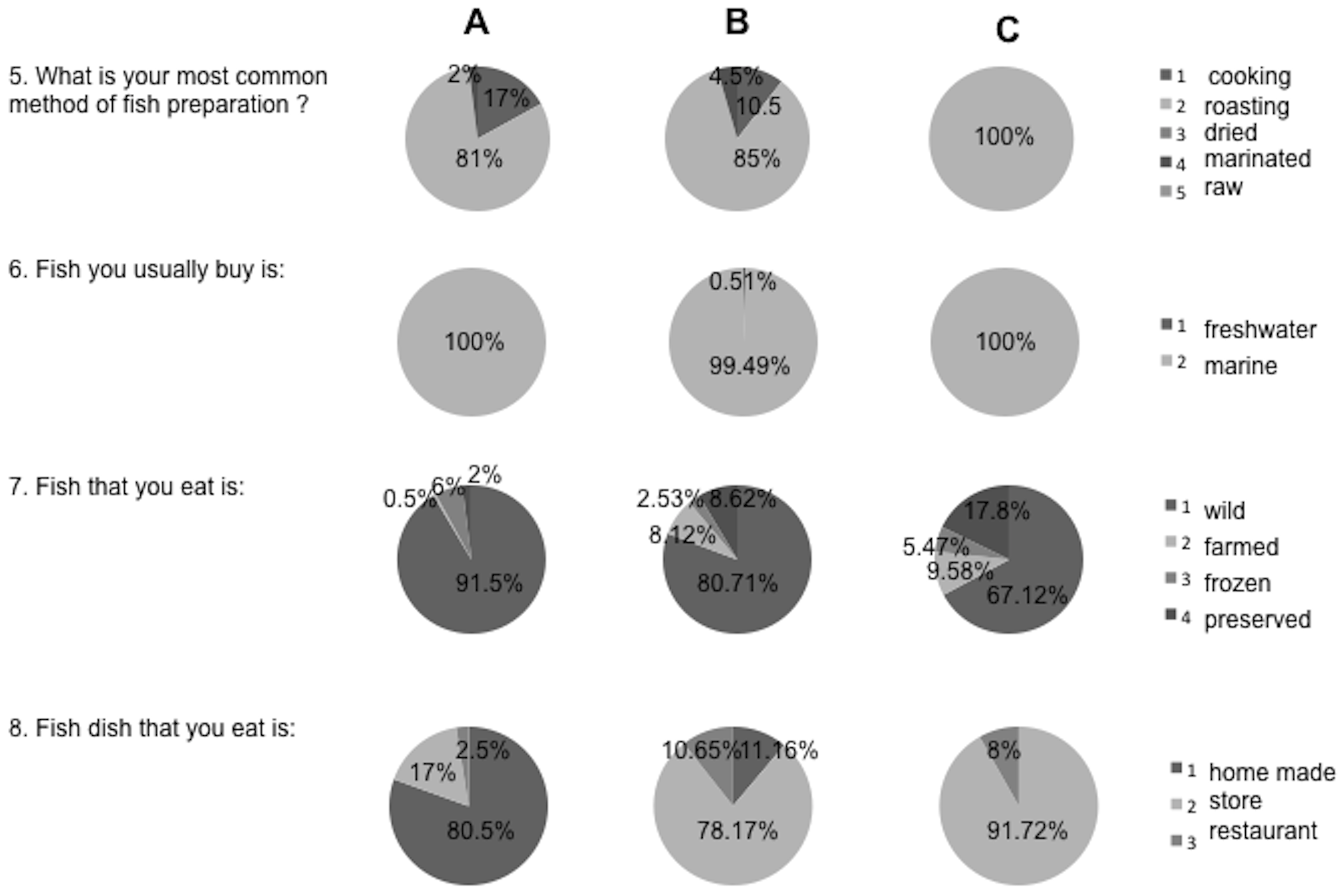

Supplement: Figure S2 — Questionnaire about fish cooking habits. A: islands population; B: coastal urban population; C: inland rural population. (TIFF) [file pntd.0002673.s002.tiff]
