# Supplementary material for: Anti-Anisakis IgE Seroprevalence in the Healthy Croatian Coastal Population and Associated Risk Factors
Source: PLoS Negl Trop Dis. 2014 Feb 6;8(2):e2673. doi: 10.1371/journal.pntd.0002673 (PMC3916232; doi:10.1371/journal.pntd.0002673)
Supplement: Text S1 — Questionnaire layout used in this study. (DOC) [file pntd.0002673.s003.doc]

**QUESTIONNAIRE (supplement file S3)**

Personal data

County:_________________________________________________

1. Date of birth ________________________
2. Place of living/ residence ______________
3. Marital status: a) married

b) single

c) widow/widower

d) divorced

e) common-law marriage

*(Please circle or specify only one answer to the following questions)*

1. What is the highest level of education you have completed?
2. without finishing primary school
3. completed primary school
4. handcraft or industrial school
5. high school
6. faculty (university or high school, academy)
7. postgraduate studies, doctorate
8. other (please specify in more detail) __________________________
9. Employment status: a) employed

b) not employed

c) temporarily incapable for work (sick-leave)

1. Of which chronic diseases do you suffer?
2. lung diseases
3. heart diseases
4. digestive diseases
5. allergic diseases (atopic dermatitis, asthma and rhinitis)
6. malignancies
7. other __________________________________
8. Are you fishermen or do you work in the fishing industry?
   1. Yes
   2. No

Eating habits

1. Do you consume meat (animal origin)?
2. Yes
3. No
4. Do you consume fish and fish products?

a) Yes

b) No

1. Do you consume thermally untreated fish (raw, salted, marinated, sushi)
2. Yes
3. No
4. How often do you eat fish and fish products?
5. everyday
6. several times a week
7. once a week
8. rarely
9. never

1. What is your most common method of fish preparation?
2. cooking
3. roasting
4. dried
5. marinated
6. raw
7. Fish you usually buy is:
8. freshwater
9. marine
10. Fish that you eat is:
11. wild
12. farmed
13. frozen
14. preserved
15. Fish dish that you eat is:
    a) homemade
    b) from stores
    c) from restaurant

In ____________, ________ 2011.
